# Clinical, thermographic, and tensiometric evaluation of rat cutaneous wounds treated with collagen gel associated with F18 bioactive glass

**DOI:** 10.1590/acb399424

**Published:** 2024-11-29

**Authors:** Bruna Martins da Silva, Ivan Felismino Charas dos Santos, Paula Mancuso, Letícia Albuquerque Fortes Pereira, Ivan Moroz, Marina Frazatti Gallina, Miriam Harumi Tsunemi, Marina Trevelin Souza, Claudia Helena Pellizzon, José Ivaldo Siqueira Silva, Cláudia Valéria Seullner Brandão, Liandra Amara Garcia Alves

**Affiliations:** 1 Universidade Estadual Paulista – School of Veterinary Medicine and Animal Science – Postgraduate Program in Animal Biotechnology – Botucatu (SP) – Brazil.; 2 Universidade Federal de Rondônia – Rolim de Moura (RO) – Brazil.; 3 Universidade Estadual Paulista – School of Veterinary Medicine and Animal Science – Botucatu (SP) – Brazil.; 4 Universidade Estadual Paulista – School of Agronomic Sciences – Department of Bioprocesses and Biotechnology – Botucatu (SP) – Brazil.; 5 Universidade Estadual Paulista – Institute of Biosciences of Botucatu – Department of Biostatistics – Botucatu (SP) – Brazil.; 6 Vetra Pesquisa e Desenvolvimento de Produtos Cerâmicos de Alta Tecnologia – Ribeirão Preto (SP) – Brazil.; 7 Universidade Estadual Paulista – Institute of Biosciences of Botucatu – Department of Structural and Functional Biology – Botucatu (SP) – Brazil.; 8 Universidade Estadual Paulista – School of Veterinary Medicine and Animal Science – Department of Veterinary Surgery and Animal Reproduction – Botucatu (SP) – Brazil.

**Keywords:** Biocompatible Materials, Materials Testing, Wound Healing, Skin, Collagen

## Abstract

**Purpose:**

To evaluate the association of collagen gel with F18 bioactive glass (BG) in the healing of non-contaminated cutaneous wounds induced in healthy Wistar rats.

**Methods:**

One hundred twelve adult and healthy Wistar rats were randomly divided into four groups (n = 28): saline solution (0.9%); healing ointment based on allantoin and zinc oxide; collagen gel; and association of F18 BG powder and collagen gel. All the rats underwent the creation of a 3-cm diameter wound in their dorsal region. Macroscopic, thermographic, and tensiometric evaluations of the wound were performed.

**Results:**

The presence of granulation tissue varied significantly in and between the groups. The surface temperature assessed through thermography of wounds treated with saline solution (0.9%) increased significantly over time and between the groups. No difference was identified regarding tensiometry.

**Conclusions:**

Collagen gel associated with F18 BG induced beneficial healing effects on non-contaminated cutaneous wounds in Wistar rats, which included the induction of increased blood perfusion as assessed through thermography.

## Introduction

Cutaneous wounds are among the most common conditions in veterinary clinical-surgical practice. Consequently, there is ongoing research into products that are cost-effective, promote faster healing with stronger scar tissue, and have minimal side effects and no environmental contaminants^1^^,^ 2. In this context, bioglass is a type of biomaterial increasingly used in tissue regeneration due to its biological inertness and ability to form interfacial bonds with surrounding living tissue^3^. This property provides an initial support structure for cellular organization or stimulates cellular activity during the healing proces^3^. During degradation, bioglass releases calcium, sodium, silicon, and phosphate, which contribute to tissue regeneration^3^^,^ 4. Additionally, bioglasses possess other beneficial properties, including osteoinduction, angiogenesis, anti-inflammatory and antibacterial actions, vasodilation, and cell modulation^3^^,^ 5 ^–^ 11.

F18 bioactive glass (BG) has demonstrated biocompatibility and biofunctionality, along with the capability to be produced in various forms such as fibers, powders, and scaffolds^12^. Additionally, it is a biomaterial composed of silicon dioxide (SiO2), sodium dioxide (Na2O), potassium dioxide (K2O), magnesium oxide (MgO), calcium oxide (CaO), and phosphorus pentoxide (P2O5), which ensure controlled crystallization during prolonged or repetitive thermal treatments, unlike other bioglasses^12^.

Research on the use of F18 BG in cutaneous lesions is scarce in literature. A pilot study conducted on burn wounds in rats showed that F18 BG had beneficial effects, such as faster healing, stimulation of fibroblast and keratinocyte production, connective tissue formation, and an increase in vascular endothelial growth factor (VEGF)^12^. However, to date, no studies have evaluated the effects of F18 BG on the healing of surgically induced cutaneous wounds, leaving a gap in understanding how this biomaterial influences the healing process. This highlights the novelty of the present study.

Consequently, this study aimed to evaluate the effects of collagen gel associated with F18 BG on the healing of non-contaminated cutaneous wounds induced in healthy Wistar rats, using clinical, thermographic, and tensiometric analyses. The hypothesis was that F18 BG would demonstrate beneficial effects on cutaneous wound healing compared to wounds treated with saline solution (0.9%), healing ointment, and collagen gel alone.

## Methods

### Animals and experimental design

This study was approved by the institutional Ethics Committee for the Use of Animals (number 0074/2021).

One hundred twelve healthy male Wistar rats, *Rattus norvegicus*, heterogenic, aged between 8 and 10 weeks old, with a mean body mass between 365 and 370 g, were used. The animals were housed at the Experimental Unit of the Department of Veterinary Surgery and Animal Reproduction of School of Veterinary Medicine and Animal Science (Botucatu, São Paulo, Brazil) (GPS: S 22°53’17.5 WO 48°29’55.4).

The rats were subjected to acclimatization in the experimental environment for 10 days, in groups of four animals in polysulfone plastic boxes (length = 497 mm, width = 341 mm, height = 265 mm). The animals received a commercial pelleted diet and filtered water *ad libitum*, and the environment remained air-conditioned, with temperature control (23 ± 1°C), humidity varying between 40 and 60%, and 12-hour light/dark cycles. Deworming with ivermectin 1% (0.1 mL diluted in 100 mL of drinking water from a collective water fountain) was carried out, every seven days, for a total of 14 days.

After 10 days of acclimatization, the animals were placed individually in another polysulfone boxes (length = 385 mm, width = 251 mm, height = 240 mm), and crumpled bond paper was used as environmental enrichment. These boxes were cleaned every 48 hours with water and neutral soap.

### Anesthesia and surgical procedure of the ischemic skin flaps

For the creation of the cutaneous wounds, the rats were premedicated with morphine sulfate pentahydrate (2 mg/kg), administered subcutaneously (SC), and anesthetized with a combination of ketamine hydrochloride (75 mg/kg) and xylazine (10 mg/kg) administered intraperitoneally (IP).

**Figure 1:**
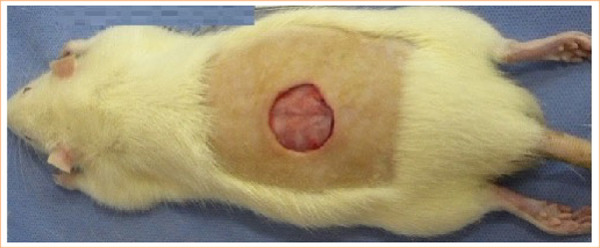
A photograph image showing a 3-cm diameter wound after the skin and subcutaneous fat were removed, revealing the underlying muscle fascia.

Source: Elaborated by the authors.

The rats were placed in ventral recumbency, and after manual epilation of the dorsal region followed by skin asepsis with 2% chlorhexidine, a 3-cm diameter wound was created using a sterile metal punch in the middle third of the distance between the atlanto-occipital region and the base of the tail. The skin and subcutaneous fat were removed using sterile Metzenbaum scissors and anatomical forceps, while preserving the muscle fascia (Fig. 1).

For postoperative pain control, tramadol hydrochloride 2% (10 mg/kg, SC) was administered every 12 hours for 48 hours. The wounds were left uncovered, without bandages. No antibiotics or anti-inflammatory drugs were administered during the entire study period. The surgical procedures were conducted by the same experienced surgeon at 5 a.m., following the asepsis protocols.

### Treatments

After the skin wounds were created, the rats were randomly assigned to four groups (n = 28), using the Randomizer program (2018), based on the treatment protocol:

- Group 1 (G1): 0,1 mL of saline solution 0.9% every 24 hours;
- Group 2 (G2): 0.1 g of healing ointment based on allantoin and zinc oxide every 24 hours;
- Group 3 (G3): 2 mL of collagen gel every 72 hours;
- Group 4 (G4): association of F18 BG powder (200 mg) and collagen gel (2 mL) every 72 hours^12^.

The treatments began immediately after the wounds were created and continued for 21 days. The wounds were superficially cleansed using sterile gauze moistened with saline solution 0.9%, without removing the crust.

The collagen gel and F18 BG powder were provided by the Laboratory of Glass Materials, Universidade Federal de São Carlos, São Carlos, São Paulo, Brazil. The F18 BG composition included SiO_2_, Na_2_O, K_2_O, MgO, CaO, and P_2_O_5_ (patent BR10 INPI 20130209619). The BG powder was prepared according to the literature, with a particle size of 49 μm in diameter.

The collagen gel was produced by mixing collagen with glycerin and water in a 1:5 ratio, with glycerin and water volumes combined in a 4:3 ratio. pH changes over time were monitored using a phosphate-buffered saline solution with a weight/volume ratio of 50 mg/mL

### Clinical evaluation

The clinical evaluation included body mass (BM) and body temperature (BT) measurements, as well as macroscopic evaluation of the wounds. BM was measured using a precision digital scale, and BT was determined with infrared thermography to minimize stress and avoid alterations in the results. Measurements were consistently taken at the same time (7 a.m.) in a room with blackout curtains, a constant temperature of 21 ± 2°C, and humidity between 40 and 50%, with only three people present.

**Figure 2:**
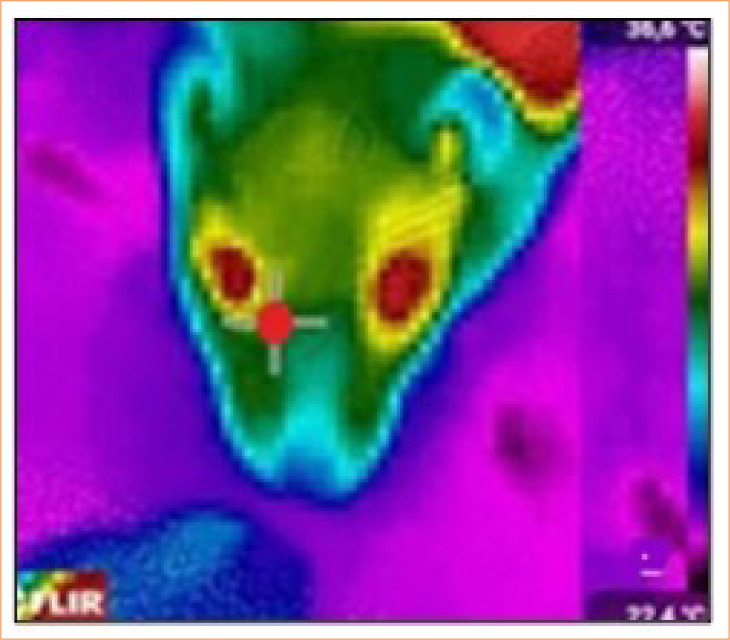
Thermographic image of the Wistar rat’s head, highlighting the right lacrimal caruncle region (red dot), used to measure surface temperature as body temperature through infrared thermography.

Source: Elaborated by the authors.

For infrared thermography measurements, the rats were physically restrained to minimize stress and positioned in ventral recumbency. Thermographic images were captured using an infrared thermograph (FLIR Model E4) from 10 cm, perpendicular to the right lacrimal caruncle (Fig. 2). The superficial temperature of the region was determined using a Flir software. All assessments were conducted by the same evaluator.

Crust, granulation tissue, and wound contamination were assessed through clinical observation (macroscopic evaluation) and classified based on the presence or absence of these alterations, as a semiquantitative classification.

The BM and BT were evaluated in the following time points: 10 minutes before the creation of the skin wounds (M0), and three days (M3d), seven days (M7d), 14 days (M14d), and 21 days (M21d) after the creation of the skin wounds. The presence of crust, granulation tissue, and wound contamination was determined at same time points, except at M0.

### Wounds thermographic evaluation

**Figure 3:**
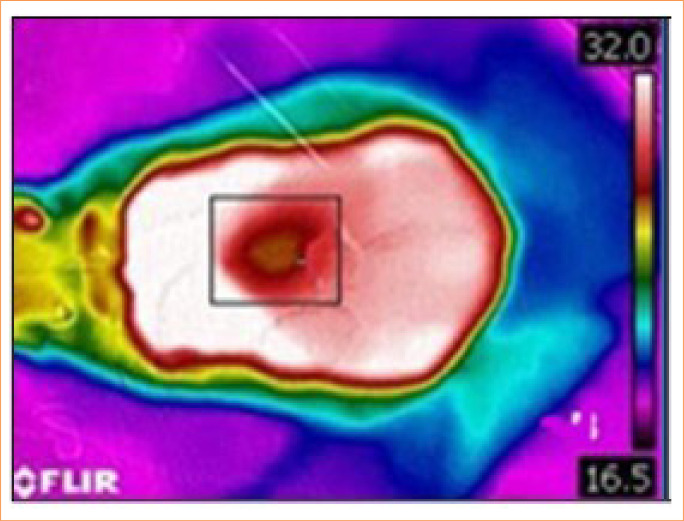
Thermographic image illustrating the wound, with the surface temperature measurement area highlighted (black rectangle) using infrared thermography.

Source: Elaborated by the authors.

The thermographic evaluation of the wounds followed the same methodology as the infrared thermographic assessment of the right lacrimal caruncle region. However, during this process, the rats were physically restrained in ventral recumbency, ensuring that the wounds remained undisturbed. Thermographic images were taken from a distance of 10 cm, perpendicular to the wound area (Fig. 3).

The evaluations were carried out in the following time points: 15 minutes (M15min) after the creation of the skin wounds, M3d, M7d, M14d, and M21d.

### Tensiometric evaluation

The rats of each group were randomly divided into four groups of seven animals each and euthanized using a combination of xylazine 2% (30 mg/kg) and ketamine hydrochloride 1% (180 mg/kg) (IP) at the following time points: M3d, M7d, and M14d.

After euthanasia, excisional biopsies of the wounds were performed with a 3-cm margin of intact skin around the lesion, down to the muscle fascia. Then, specimens containing the wound and at least 2 cm of healthy tissue at each end were prepared.

**Figure 4:**
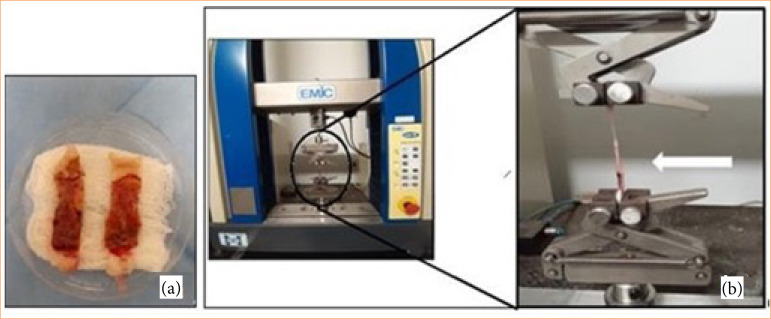
Image illustrating the wound specimen prepared for tensiometric evaluation: **(a)** fixed in the universal mechanical testing machine (EMiC) (white arrow) using clamps, with 1 cm on each side from the sample edge, leaving a 1 cm free area from the wound; **(b)** close-up of the clamped area and the free section of the wound specimen.

Source: Elaborated by the authors.

The specimens were fixed in a universal mechanical testing machine (EMiC) using clamps (Figs. 4a and 4b), and subjected to a mechanical test in the vertical direction with a constant traction speed of 20 mm/second and load cell range of 1 to 50 N.

The maximum tensile strength (MTS) and maximum deformation (MD) values were recorded in the specific software (Mtest). The evaluations were carried out by a professional experienced in mechanical testing of biomaterials.

### Statistical analysis

Statistical analyses were conducted using the R software (version 4.2.0, 2022). The normality of the variables at each moment and group was assessed by the Shapiro-Wilk’s test. To compare the groups at each moment and the moments within each group, the one-way analysis of variance (ANOVA) was used for normal data and the Kruskal-Wallis test for non-normal variables. To compare the BM between the induction moment and the euthanasia moments for each group, the Friedman test was used. Differences with *p* < 0.05 were considered significant.

## Results

No significant differences in BM and BT were observed (Tables 1 and 2).

**Table 1:** Mean and standard deviation of body mass **(g)** in Wistar rats with wounds treated with saline solution (0.9%) (G1), healing ointment based on allantoin and zinc oxide (G2), collagen gel (G3), and a mixture of F18 bioglass powder and collagen gel (G4), evaluated at the moments: before anesthesia induction (M0); three days (M3d), seven days (M7d), 14 days (M14d), and 21 days (M21d) after wound induction*.

Groups	M0	M3d	M7d	M14d	M21d
G1	410.3 ± 52.1^Aa^	408.5 ± 41.9^Aa^	405.5 ± 68.0^Aa^	425.2 ± 48.5^Aa^	435.2 ± 41.5^Aa^
G2	409.3 ± 41.8^Aa^	374.0 ± 32.2^Aa^	361.7 ± 19.5^Aa^	381.5 ± 37.0^Aa^	411.2 ± 31.7^Aa^
G3	397.5 ± 41.3^Aa^	399.1 ± 31.9^Aa^	429.7 ± 29.7^Aa^	391.3 ± 32.2^Aa^	397.3 ± 26.1^Aa^
G4	367.9 ± 37.4^Aa^	368.4 ± 43.9^Aa^	361.8 ± 21.4^Aa^	373.1 ± 40.1^Aa^	380.8 ± 34.4^Aa^

* Means followed by different uppercase letters in the same column were significantly different by the analysis of variance test (*p* < 0.05).

Means followed by different lowercase letters in the same row were significantly different by the analysis of variance test (*p* < 0.05). Source: Elaborated by the authors.

**Table 2:** Mean and standard deviation of body temperature values (ºC) through infrared thermography, in the right lacrimal caruncle region of Wistar rats with wounds treated with saline solution (0.9%) (G1), healing ointment based on allantoin and zinc oxide (G2), collagen gel (G3), and a mixture of F18 Bioglass powder and collagen gel (G4), evaluated at the moments: before anesthesia induction and wound creation (M0); three days (M3d), seven days (M7d), 14 days (M14d), and 21 days (M21d) after wound induction*.

Groups	M0	M3d	M7d	M14d	M21d
G1	38.54 ± 0.81^Aa^	38.30 ± 0.95^Aa^	38.16 ± 0.36^Aa^	38.16 ± 0.36^Aa^	38.54 ± 0.81^Aa^
G2	38.54 ± 0.54^Aa^	38.81 ± 0.61^Aa^	38.91 ± 1.21^Aa^	38.64 ± 1.35^Aa^	38.54 ± 0.54^Aa^
G3	38.91 ± 1.42^Aa^	38.44 ± 1.30^Aa^	38.51 ± 1.26^Aa^	38.59 ±1.26^Aa^	38.92 ± 1.42^Aa^
G4	38.57 ± 0.80^Aa^	38.09 ± 0.99^Aa^	38.20 ± 0.78^Aa^	38.20 ± 0.43^Aa^	38.57 ± 0.80^Aa^

* Means followed by different uppercase letters in the same column were significantly different by the analysis of variance test (*p* < 0.05).

Means followed by different lowercase letters in the same row were significantly different by the analysis of variance test (*p* < 0.05). Source: Elaborated by the authors.

Wounds treated with saline solution (0.9%) and healing ointment showed a significant decrease (*p* < 0.05) in crust intensity between M3d and M21d, and between M7d and M21d (Table 3). In the comparison between groups, wounds in G1 exhibited significantly higher crust intensity (*p* < 0.05) compared to those in G3 and G4 at all time points, except M21d (Table 3).

**Table 3:** Macroscopic variables (crust, granulation tissue, and contamination) of the wounds treated with saline solution (0.9%) (G1), healing ointment based on allantoin and zinc oxide (G2), collagen gel (G3), and mixture of F18 Bioglass powder and collagen gel (G4), evaluated three days (M3d), seven days (M7d), 14 days (M14d), and 21 days (M21d) after wound induction*.

Variables/Groups	M3d	M7d	M14d	M21d
Crust				
G1	3^ABa^	3^Aa^	2^ABab^	1^Ab^
G2	4^Aa^	4^Aa^	3^Bab^	2^Ab^
G3	2^Ba^	1^Ba^	1^Aa^	1^Aa^
G4	3^ABa^	3^Aa^	1^Ab^	1^Ab^
Granulation tissue				
G1	3^Aa^	3^Aa^	2^Aa^	2^ABa^
G2	1^Ba^	1^Ba^	3^Ab^	3^Aab^
G3	4^Aa^	2^ABb^	2^Ab^	2^Bb^
G4	1^Bb^	3^Aa^	2^Aab^	1^Bb^
Contamination				
G1	2^Aa^	1^Aa^	1^Aa^	1^Aa^
G2	2^Aa^	1^Aa^	1^Aa^	1^Aa^
G3	2^Aa^	1^Aa^	1^Aa^	1^Aa^
G4	1^Aa^	1^Aa^	1^Aa^	1^Aa^

* Classifications followed by different uppercase letters in the same column were significantly different by the analysis of variance test (*p* < 0.05).

Means followed by different lowercase letters in the same row were significantly different by the analysis of variance test (*p* < 0.05). Classifications of the variables crust and granulation tissue: absent (degree 1), mild (degree 2), moderate (degree 3), and severe (degree 4). Classifications of the variable contamination: absent and present. Source: Elaborated by the authors.

Regarding the granulation tissue variable, a significant increase (*p* < 0.05) was observed, from absent to moderate, in wounds treated with healing ointment between M3d and M14d, M3d and M21d, M7d and M14d, and M7d and M21d.

Wounds treated with collagen gel showed a significant decrease (*p* < 0.05), from severe to mild or absent, between M3d and all subsequent time points (Table 3). In G4, a statistically significant increase (*p* < 0.05) was observed, from absent to moderate, between M3d and M7d, followed by a significant decrease (*p* < 0.05), from moderate to absent, between M7d and M21d (Table 3).

**Figure 5:**
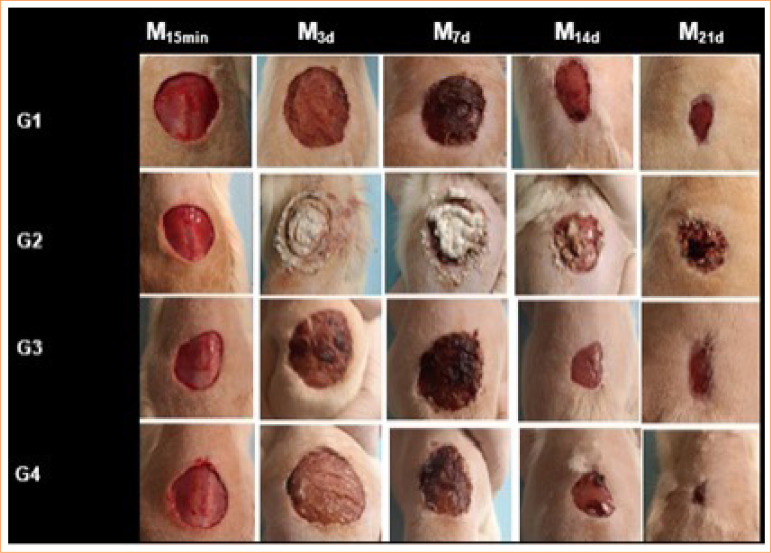
Macroscopic healing progression of the wounds over the study period. G1: wounds treated with saline solution (0.9%); G2: healing ointment based on allantoin and zinc oxide; G3: collagen gel; and G4: association of F18 Bioglass powder and collagen gel, evaluated at the moments: 5 minutes before created skin wound (M15min); three days after created skin wound (M3d); seven days after created skin wound (M7d); 14 days after created skin wound (M14d); and 21 days after created skin wound (M21d).

Source: Elaborated by the authors.

In the comparison between groups, wounds in G1 and G3 had significantly higher granulation tissue intensity (*p* < 0.05) compared to those in G2 and G4 at M3d and M7d. Wounds in G1 and G4 showed significantly higher granulation tissue intensity (*p* < 0.05) compared to those in G2 (Table 3). Finally, at M14d, wounds in G1 and G2 exhibited statistically higher granulation tissue intensity (*p* < 0.05), ranging from mild to moderate, compared to those in G3 and G4, which showed no granulation tissue (Table 3). The progression of the healing process is illustrated in Fig. 5.

Regarding the infrared thermographic evaluation of the wounds, a significant increase (*p* < 0.05) in surface temperature values was observed in wound beds treated with saline solution (0.9%) between M3d and M7d (Table 4). In the comparison between groups at M3d, significantly higher temperatures (*p* < 0.05) were found in all wounds except those in G1 (Table 4).

**Table 4:** Mean and standard deviation of the surface temperature values of the cutaneous wound bed (ºC), measured using infrared thermography, in rats with wounds treated with saline solution (0.9%) (G1), healing ointment based on allantoin and zinc oxide (G2), collagen gel (G3), and mixture of F18 bioglass powder and collagen gel (G4), at the moments: 15 minutes (M15min), three days (M3d), seven days (M7d), 14 days (M14d), and 21 days (M21d) after wound induction*.

Groups	M15min	M3d	M7d	M14d	M21d
G1	38.51 ± 0.31^Aa^	38.66 ± 0.36^Aa^	38.10 ± 0.69^Ab^	38.00 ± 0.10^Aab^	38.00 ± 0.14^Aab^
G2	38.24 ± 0.24^Aa^	38.47 ± 0.17^Ba^	38.07 ± 0.17^Aa^	38.56 ± 0.56^Aa^	38.94 ± 0.79^Aa^
G3	38.11 ± 0.41^Aa^	38.38 ± 0.46^Ba^	37.10 ± 0.12^Aa^	37.61 ± 0.12^Aa^	37.23 ± 0.25^Aa^
G4	38.57 ± 0.80^Aa^	38.40 ± 0.66^Ba^	38.11 ± 0.11^Aa^	38.60 ± 0.12^Aa^	38.07 ± 0.14^Aa^

* Means followed by different uppercase letters in the same column were significantly different by the analysis of variance test (*p* < 0.05).

Means followed by different lowercase letters in the same row were significantly different by the analysis of variance test (*p* < 0.05) Source: Elaborated by the authors.

In the tensiometric evaluation, no significant variations were detected in the comparisons between or in the groups regarding MTS and MD (Table 5).

**Table 5:** Mean and standard deviation of the maximum tensile strength (MTS) and maximum deformation (MD) of Wistar rat cutaneous wounds treated with saline solution (0.9%) (G1), healing ointment based on allantoin and zinc oxide (G2), collagen gel (G3), and mixture of F18 bioglass powder and collagen gel (G4), evaluated three days (M3d), seven days (M7d), 14 days (M14d), and 21 days (M21d) after wound induction*.

Groups/Variables	M3d	M7d	M14d
MTS (N)			
G1	0.29 ± 0.21^Aa^	0.22 ± 0.19^Aa^	0.19 ± 0.15^Aa^
G2	0.17 ± 0.14^Aa^	0.10 ± 0.09^Aa^	0.17 ± 0.08^Aa^
G3	0.19 ± 0.18^Aa^	0.22 ± 0.24^Aa^	0.17 ± 0.11^Aa^
G4	0.12 ± 0.14^Aa^	0.17 ± 0.09^Aa^	0.50 ± 0.14^Aa^
MD (mm)			
G1	23.93 ±18.66^Aa^	23.19 ± 21.66^Aa^	38.40 ± 31.90^Aa^
G2	20.66 ± 14.56^Aa^	22.30 ± 18.41^Aa^	24.69 ± 17.19^Aa^
G3	31.81 ± 22.32^Aa^	46.80 ± 18.08^Aa^	27.68 ± 16.14^Aa^
G4	33.85 ± 25.23^Aa^	32.01 ± 26.15^Aa^	16.70 ± 21.87^Aa^

* Means followed by different uppercase letters in the same column were significantly different by the analysis of variance test (*p* < 0.05).

Means followed by different lowercase letters in the same row were significantly different by the analysis of variance test (*p* < 0.05). Source: Elaborated by the authors.

## Discussion

The healing effect of collagen gel associated with F18 BG in surgically induced wounds in Wistar rats was studied due to the scarcity of research on the use of this bioglass in wound healing^12^. To date, there are no known studies related to the use of F18 BG in non-contaminated cutaneous wounds, which underscores the novelty of this research. However, earlier studies have demonstrated several beneficial properties of F18 BG, including its bactericidal, angiogenic, and vasodilation effects, as well as its biocompatibility and ability to stimulate bone tissue formation and joint regeneration^5^^–^ 7 ^,^ 10 ^,^ 13 ^,^ 14.

Previous investigations with hydroxyapatite- or calcium-based bioglasses have shown faster healing in diabetic wounds in rats^15^, as well as stimulation of collagen tissue contraction in cutaneous wounds in mice^16^. A pilot study on Wistar rats with burn-induced cutaneous lesions treated with F18 BG demonstrated faster tissue repair, increased production of fibroblasts, keratinocytes, and connective tissue, along with an increase in VEGF^12^.

The initial hypothesis was confirmed, as wounds treated with F18 BG not only exhibited the highest surface temperatures, suggesting that F18 BG induced vasodilation, but also showed the greatest MTS compared to the other groups. The evidence that F18 BG could act beneficially in the healing of cutaneous wounds is associated with its composition (SiO_2_–Na_2_O–K_2_O–MgO–CaO–P_2_O_5_), high tissue reactivity, and enhanced biointeraction, which stimulates cellular proliferation and the regeneration of both hard and soft tissues^12^^,^ 17. The biocompatibility of F18 BG was evaluated *in vivo* through subcutaneous implantation in rats, with subsequent evaluation over 60 days^17^. Histological analysis revealed that F18 BG had beneficial effects on tissue, including improved capsule thickness and quality, as well as a favorable bioglass-tissue interface. No rejection reaction was observed, and the degraded material was absorbed^17^.

The BT measured via infrared thermography at the lacrimal caruncle showed no significant variation and was associated with the absence of wound infection. Measuring BT using infrared thermography is considered a recent methodology in the field^18^^,^ 19, reducing the likelihood of stress to animals compared to rectal temperature measurement with a digital thermometer^19^. Additionally, measuring BT at the lacrimal caruncle region through infrared thermography is associated with the high concentration of capillaries in the area, which are controlled by the autonomic nervous system for body heat dissipation^18^. Zanghi^19^ correlated the temperature of the lacrimal caruncle and ear regions, measured via infrared thermography, with rectal temperature, and observed that the surface temperature of the lacrimal caruncle, measured by infrared thermography, had a positive correlation with rectally measured temperature.

At the beginning of treatment, contamination was identified in all groups except the one treated with the association of collagen gel and F18 BG powder. However, after three days of treatment, no signs of contamination were detected. This initial contamination was associated with the proliferation of the skin microbiota, which was previously in symbiosis. It is worth noting that F18 BG exhibits bactericidal action, which is linked to increased local pH, as well as changes in osmotic pressure and electrostatic forces^6^^,^ 7 ^,^ 10 ^,^ 12 ^,^ 13.

The wounds treated with saline solution (0.9%) showed significantly higher crust intensity compared to those treated with collagen gel and its mixture with F18 BG powder at all moments, except at Md21. Besides being formed by cellular debris, the crust ensures protection to the wound, and it has been postulated that the fibroplasia process can be stimulated by crust presence^20^.

The absence of granulation tissue in the group treated with the association of collagen gel and F18 BG powder at M21d was attributed to the healing properties of the biomaterial, which include the increased production of growth factors responsible for cell proliferation and vasodilation^5^^,^ 12. This finding demonstrated the beneficial effect of F18 BG on the healing of cutaneous wounds.

The evaluation of wound surface temperature aimed to identify vascular changes and inflammatory responses, as high temperatures can indicate vasodilation and increased blood perfusion^21^, serving as a possible alternative to laser Doppler. In the present study, the significantly lower surface temperature values of the wound treated with saline solution 0.9% compared to the other groups at M3d indicated reduced blood perfusion in the wounds. It is important to note that this period corresponded to the inflammatory phase, characterized by vasodilation and increased blood perfusion^20^^,^ 22. However, wounds treated with the association of collagen gel and F18 BG showed higher surface temperature values, although without significant differences, likely due to the stimulation of nitric oxide (NO) release, an endogenous vasodilator induced by F18 BG^5^. Conversely, Gillette et al.^23^ assessed blood perfusion in non-contaminated wounds in dogs treated with ceramic biomaterial using laser Doppler. The authors found no significant differences in blood perfusion between the experimental and control groups, neither three nor five days after wound induction.

The absence of significant variation in tensiometry was consistent with the literature^23^. Although no significant difference was observed in MTS and MD values, an increase in rupture resistance was noted in wounds treated with F18 BG. These wounds reached higher MTS values compared to the other groups and showed a decrease in MD.

## Conclusion

F18 BG has beneficial effects on the healing of non-contaminated, surgically induced cutaneous wounds in Wistar rats, including the induction of increased blood perfusion, as evidenced by the rise in the surface temperature of the wound.

## Data Availability

All data were generated or analyzed in the current study.
